# Is sexual risk taking behaviour changing in rural south-west Uganda? Behaviour trends in a rural population cohort 1993–2006

**DOI:** 10.1136/sti.2008.033928

**Published:** 2009-03-13

**Authors:** S Biraro, L A Shafer, I Kleinschmidt, B Wolff, A Karabalinde, A Nalwoga, J Musinguzi, W Kirungi, A Opio, J Whitworth, H Grosskurth

**Affiliations:** 1 Medical Research Council (MRC)/Uganda Virus Research Institute (UVRI) Uganda Research Unit on AIDS, Entebbe, Uganda; 2 London School of Hygiene and Tropical Medicine, London, UK; 3 Ministry of Health, Government of Uganda; 4 Wellcome Trust

## Abstract

**Objective:**

To describe sexual behaviour trends in a rural Ugandan cohort in the context of an evolving HIV epidemic, 1993–2006.

**Methods:**

Sexual behaviour data were collected annually from a population cohort in which HIV serological surveys were also conducted. Behaviour trends were determined using survival analysis and logistic regression. Trends are reported based on the years in which the respective indicators were collected.

**Results:**

Between 1993 and 2006, median age at first sex increased from 16.7 years to 18.2 years among 17–20-year-old girls and from 18.5 years to 19.9 years among boys. Both sexes reported a dip in age at sexual debut between 1998 and 2001. One or more casual partners in the past 12 months among men rose from 11.6% in 1997 to 12.7% in 2004 and then declined to 10.2% in 2006. Among women it increased from 1.4% in 1997 to 3.7% in 2004 and then reduced to 1.4% in 2006. The rise in casual partners between 1997 and 2004 was driven mainly by older age groups. Trends in condom use with casual partners varied by age, increasing among those aged 35+ years, declining in the middle age groups and presenting a dip and then a rise in the youngest aged group (13–19 years).

**Conclusion:**

Among youth, risky behaviour declined but increased in the late 1990s/early 2000s. Among those aged 35+ years, condom use rose but casual partners also rose. Several indicators portrayed a temporary increase in risk taking behaviour from 1998 to 2002.

**Take-home messages**

- The changes in sexual behaviour in this cohort were not consistent across the variables and subgroups investigated.
- Some indicators showed encouraging trends toward safer sexual behaviour, particularly among young people.
- Other indicators suggest an increase in sexual risk taking, particularly among middle-aged and older adults.
- These changes may help explain recent changes in epidemiological trends in Uganda, but more work is required to establish such links if they exist.

In the context of the HIV/AIDS epidemic, the carefully conducted documentation of population trends in self-reported sexual behaviour is required for a number of reasons. Perhaps the most important is that data on behavioural changes can alert policymakers and intervention programme managers of developments that necessitate the adaptation of ongoing intervention strategies. Second, trends in reported behaviour can help to interpret observed epidemiological changes in HIV infection and other sexually transmitted infections. Through comparative analyses, they also help to better understand differences in the course of the HIV epidemic between countries; they may be useful in feeding mathematical models designed to predict the course of the epidemic and the most cost-effective intervention measures. Lastly, behavioural data are also of interest to anthropological research in their own right.

In Uganda, data on sexual behaviour and their trends over time are available from national surveys, demographic and health surveys, sentinel surveillance groups and from a few longitudinal cohort studies accompanied by qualitative surveys.^1^^–^5 National cross-sectional surveys and data from sentinel groups such as antenatal clinic attenders should be supplemented by longitudinal data gathered from annual surveys of well characterised cohorts. Such data refer to a smaller geographical area, but any trends observed are related to an identical study population and can offer more detailed insights, particularly if combined with qualitative behavioural research conducted in the same community.

Several studies have documented changes in sexual behaviour in Uganda leading to a decrease in risk taking during the 1990s, and various researchers have used these observations to explain the declines observed in the HIV epidemic during that period.^2^ ^3^ ^6^^–^9 We have previously documented sexual behaviour as well as HIV prevalence and incidence in a rural population cohort from south-west Uganda that has been studied since 1989.^3^ ^10^ ^11^ Here we provide an update on trends in self-reported sexual behaviour in this cohort, covering the period from 1997 to 2006.

According to guidelines from the World Health Organization (WHO) and the Joint United Nations Programme on HIV/AIDS (UNAIDS) for the second-generation surveillance of the HIV epidemic including sexual behaviour, three indicators have been suggested as essential for behavioural surveillance:^12^ age at sexual debut, number of sexual partners and condom use. In this paper we combine information on these indicators with additional important parameters such as secondary abstinence, pregnancy among unmarried participants, as well as the lag time between sexual debut and first marriage.

## METHODS

### Study setting

The general population cohort studied by the MRC Unit in Uganda has been described before.^3^ ^13^ ^14^ Briefly, this open cohort is located in a rural sub-county in the south-west of the country. Since 1989 the MRC has carried out annual population-based surveys in this cohort. In a private setting, survey teams administer questionnaires to those aged ⩾13 years on sexual behaviour and other factors and collect a blood sample for HIV testing. The study area, which initially comprised 15 villages, was expanded in 1999/2000 to 25 villages and now has a population of about 18 000 residents. The inclusion criterion for analysis in this study was age ⩾13 years. Voluntary counselling and testing has been available from the project counsellors since inception of the study in 1989/90. However, uptake has been low (<10% in the first 10 rounds).^15^ Among individuals aged 15–59 years, approximately 30% and 35% had ever received HIV test results by rounds 15 and 16 (survey years 2003 and 2004), respectively. At the baseline survey, polygamy among men was 7.7% and non-existent among women.

### Collection of sexual behaviour data

To reduce the burden of annual interviews on study participants, not all questions on sexual behaviour were collected at every survey round. Whether or not the respondent had ever had sex was first recorded in 1993, but age at first sex was first asked in 1997 and was then asked annually. Condom use at last sex with any partner was recorded in 1993, 1996, 1997, 1999, 2000, 2001, 2003, 2005 and 2006. Condom use at last sex with a casual partner was recorded every second year from 1997 to 2005. Casual partners were self-defined but, for those respondents who were not sure, interviewers described casual partners as those with whom the respondent had had sex once or a few times only. Information on pregnancies was obtained in most years from 1996 to 2005.

### Data management and analysis

Data were double entered and analysis was performed using Stata Version 9 (Statcorp, Texas, USA).

Two variables were used to describe sexual initiation: median age at first sex and primary abstinence, defined as the percentage of 13–19-year-olds who reported that they were not yet sexually active.

The median age at first sex was determined using survival analysis, censoring those who had not yet had sex. Persons aged 17–20 years were compared across annual survey rounds. This age range was used because it includes the median; it was intentionally narrow in order to assess trends over time with minimal overlap (the same person’s sexual debut reported in consecutive years). Trends in median age at first sex were tested using Cox survival analysis.

Secondary abstinence was defined as the percentage of persons aged 13–19 years who reported being sexually active at least once, but to have not been sexually active in the past year. For both primary and secondary abstinence, logistic regression models with a Huber correction to account for multiple observations per individual were employed to examine time trends by age and gender. Time was modelled as a linear variable with a quadratic term to allow detection of non-linear trends over time.

Regarding sexual partners, the percentage of participants reporting at least one casual partner in the past year was determined and analysed by sex, age, marital status and survey year. Logistic regression models were employed separately for men and women. In each model, age, survey year and marital status were included. Adjusted odds ratios (ORs) were calculated by adjusting for all factors in each model other than the one under consideration.

Reporting by the same individual over time in this cohort was similar but not always consistent. As an example, 53% of those who reported age at first sex in two consecutive years reported exactly the same age (or consistently reported that they had never had sex) in the 2 years. The remaining 47% of responses usually differed by 1 year and the difference went in both directions (sometimes the respondent reported an older age at sexual debut in the subsequent year and sometimes the reverse). Others have described patterns and trends in consistency of reporting,^16^ but this analysis was not within the scope of this paper. Most of the analyses in this paper treat the responses across time as though they are from a series of cross-sectional surveys; no attempt was made to validate responses by comparing those from the same individual in different survey rounds.

## RESULTS

### Sexual behaviour survey coverage

For the period 1993–9, an average of 6441 participants aged ⩾13 years took part in the census every year. For 2000–6, after 10 additional villages were included in the study area, an average of 11 194 adults participated every year. On average, 57% of these responded to the sexual behaviour questionnaire at each survey and 82% responded at least once. For survey rounds for which these data were available, lack of response was due to absence (29%) and refusal to be interviewed (13%). No systematic time trend was seen in the participation rates.

### Sexual debut, primary and secondary abstinence

Data on sexual debut were available for the period 1997–2006 and on primary and secondary abstinence for the period 1993–2006. Among participants aged 17–20 years, reported median age at first sex increased by almost a year from 17.8 years to 18.6 years between 1997 and 2006 (p for trend <0.001, survival analysis). Parallel trends were observed in girls and boys. A slight and non-significant fall in median age was recorded for both sexes between 1998 and 2000. In girls, reported median age at first sex in the years 1997–2006 was 16.7, 17.4, 17.2, 17.2, 17.5, 17.4, 17.8, 17.8, 17.9 and 18.2 years, respectively. For boys the comparative figures were 18.5, 18.0, 17.6, 17.8, 18.1, 18.6, 18.4, 18.8, 18.7 and 19.9 years.

**Figure 1:**
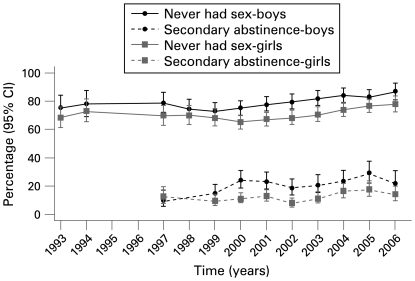
Primary and secondary abstinence in participants aged 13–19 years. Secondary abstinence is defined as no sex in the past year among those who have ever had sex.

The proportion of 13–19-year-olds who reportedly never had sex increased from 71.7% in 1993 to 82.6% in 2006 (fig 1). Although the overall trend was an increase in primary abstinence over time, both girls and boys experienced several years with a fall in the percentage who had never had sex. Among girls this fall occurred between 1999 and 2002 (p for change in trend <0.001), while among boys it was between 1998 and 2000 (p for change in trend = 0.005).

Among 13–19-year-olds who had ever had sex, there was an increase over time in those reporting no sexual partners in the past year (secondary abstinence) (fig 1). Among boys, secondary abstinence increased from 9.8% in 1997 to 28.9% in 2005 and 21.4% in 2006 (p for trend, adjusting for age = 0.001). Among girls, the comparable figures were 12.3% in 1997 and 17.4% in 2005, with a fall in 2006 to 14.0% (p for trend, adjusting for age = 0.010). Although the general trend was an increase in secondary abstinence, the non-linear trend among girls showed a temporary decline between 1997 and 2002 followed by an increase (p for change in trend = 0.038). The change in trend was of borderline significance for boys (p for change in trend = 0.078). In all age groups other than the 13–19 year age group, secondary abstinence was stable throughout the study period. Reported secondary abstinence among girls in the 13–19 year age group was about half of that observed for boys in all years except 1997. By contrast, the oldest women (age ⩾45 years) had secondary abstinence rates in the order of 65% in all years (data not shown). This was double the rate of secondary abstinence for men in this age group, which was approximately 32% in all years.

In 1997 the percentage of sexually inactive boys in the 13–19 year age group was 81% and the percentage of sexually inactive girls in this age group was 73%. Among both boys and girls, overall sexual inactivity in the past 12 months fell in 1999 and 2000 before steadily rising until 2006. By 2006 the percentage of sexually inactive boys was 90% and the percentage of sexually inactive girls was 81%.

The percentage of unmarried young women aged 13–20 years who had ever been pregnant rose from 8% in 1996 to 10% in 2003, before declining again to 8% in 2004 and 6% in 2005 (p for trend = 0.010, p for change in trend = 0.191). The percentage of unmarried women aged 21–29 years who had ever been pregnant has shown an increasing trend from 53% in 1996 to 67% in 2005 (p for trend = 0.357, p for change in trend = 0.252).

### Time from sexual debut to first marriage

The median age at first marriage among women in this cohort rose gradually from 19.0 years to 19.4 years among women aged 13–29 years between 1998 and 2006. Among men in the same age range, median age at first marriage rose from 24.9 years to 25.3 years during the same period. In 2006, 47.6% of men and 15.5% of women in the 21–29 year age group were unmarried.

For men, the median lag time between sexual debut and first marriage fell significantly between 1998 and 2006 from 7.9 years to 6.9 years (p for trend = 0.001). In contrast, for women the lag time was much shorter and showed no change over time, hovering around 2.2 years for most years between 1998 and 2006 (p for trend = 0.946).

### Casual sexual partners

Data on casual partners were available for the years 1997–2006. For this analysis, virgins were included in the category of 0 casual partners. The percentage of men reporting one or more casual partners in the past 12 months rose from 11.6% in 1997 to 12.4% in 2000 and 12.6% in 2001 and then declined to 10.7% in 2005 and 10.2% in 2006 (p for change in trend <0.001). Among women, the percentage reporting ⩾1 casual partners in the past 12 months was substantially less than among men, but increased from 1.4% in 1997 to 3.4% in 2000 and 3.6% in 2001, after which it reduced to 2.6% in 2005 and 1.4% in 2006 (p for change in trend <0.001). We also evaluated a model in which the year was entered as a categorical variable (table 1). Among both sexes, married people were less likely to report a casual partner in the past year than unmarried participants, although 9.4% of married men reported a casual partner. Among men, the percentage who reported ⩾1 casual partners in the past year declined by age group, with the exception of 13–19-year-olds who had fewer casual partners than those aged 20–24 years. Among women the trend was similar (table 1).

**Table 1:** Effects of time, age and marital status on having one or more casual partners in the past 12 months by gender, 1997–2006

Explanatory variable	Sample size*	Adjusted odds ratio (95% CI)†	p Value†
*Women*			
Age (years)			
13–19	7616 (4.0)	1 (reference)	
20–24	3188 (3.9)	1.92 (1.51 to 2.45)	<0.001
25–34	4818 (2.7)	1.54 (1.20 to 1.97)	0.001
35–44	3602 (2.5)	1.23 (0.95 to 1.60)	0.112
45+	6033 (0.8)	0.24 (0.18 to 0.34)	<0.001
Survey year			
1997	1677 (1.4)	1 (reference)	
1999	2222 (2.5)	2.01 (1.17 to 3.44)	0.012
2000	3398 (3.4)	2.98 (1.78 to 4.97)	<0.001
2001	3599 (3.6)	3.01 (1.82 to 5.00)	<0.001
2003	3535 (2.5)	2.17 (1.29 to 3.64)	0.004
2004	3805 (3.7)	3.25 (1.95 to 5.42)	<0.001
2005	3562 (2.6)	2.25 (1.33 to 3.81)	0.002
2006	3464 (1.4)	1.27 (0.73 to 2.23)	0.393
Marital status			
Unmarried	13505 (4.1)	1 (reference)	
Married	11085 (1.1)	0.19 (0.15 to 0.24)	<0.001
			
*Men*			
Age (years)			
13–19	8055 (8.2)	1 (reference)	
20–24	2241 (25.8)	4.65 (4.03 to 5.37)	<0.001
25–34	3412 (20.4)	4.66 (3.97 to 5.48)	<0.001
35–44	2514 (13.3)	2.99 (2.45 to 3.64)	<0.001
45+	4943 (4.0)	0.74 (0.60 to 0.92)	0.006
Survey year			
1997	1573 (11.6)	1 (reference)	
1999	2026 (11.1)	1.03 (0.81 to 1.30)	0.818
2000	2857 (12.4)	1.23 (0.99 to 1.52)	0.065
2001	3139 (12.6)	1.25 (1.01 to 1.54)	0.041
2003	2959 (11.6)	1.19 (0.96 to 1.48)	0.117
2004	3105 (12.7)	1.35 (1.10 to 1.68)	0.005
2005	2774 (10.7)	1.13 (0.90 to 1.41)	0.284
2006	2735 (10.2)	1.06 (0.85 to 1.33)	0.585
Marital status			
Unmarried	12579 (12.7)	1 (reference)	
Married	8031 (9.4)	0.47 (0.41 to 0.54)	<0.001

*Values in parentheses are the percentage with ⩾1 casual partners.

†Adjusted odds ratio adjusts for the factors in the models other than the one in consideration (four categorical age variables, seven year variables and one categorical variable for marital status).

‡Logistic regression Wald test with Huber correction.

**Figure 2:**
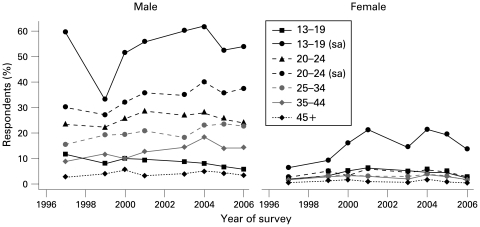
Individuals reporting one or more casual partners in the past year by age and gender of respondent. Denominator is all respondents except where indicated; sa, sexually active.

The 20–24 year age group had the highest proportion of individuals reporting ⩾1 casual sexual partners in the past year. Among men, this ranged from 23.4% in 1997 to 28.3% in 2001, 25.5% in 2005 and 24.1% in 2006 (fig 2). Among women the 13–19 year age group reported casual partners as often as the 20–24 year age group. Significant increases in respondents with ⩾1 casual partners in the past year were observed between 1997 and 2004 in several age groups, most notably the 25–34 year and 35–44 year age groups. Among 13–19-year-olds the percentage with ⩾1 casual partners declined from 1997 to 2006, but this was mainly influenced by the fact that primary and secondary abstinence among this age group was increasing (fig 1). Among sexually active individuals, reported casual partners are rising. Other than in the 13–19 year and 20–24 year age groups, the trend of reported casual partners did not differ when the denominator was sexually active individuals; nearly everybody was sexually active. By 2005 the reported number of casual partners had started to decline in all age groups.

The proportion of women reporting ⩾2 casual partners was 0.2% in 1997, 0.7% in 2001, 0.4% in 2005 and 0.2% in 2006 (p for change in trend = 0.034). Among men the percentage reporting ⩾2 casual partners in the past year was 4.6% in 1997, 6.5% in 2001, 4.7% in 2005 and 4.5% in 2006 (p for change in trend = 0.003). Although the general pattern in the cohort was an increase in those with ⩾2 casual partners until about 2001/2 and a decline thereafter, men aged 35–44 years experienced a steady incline throughout, from 4.0% in 1997 to 5.5% in 2001, 6.9% in 2005 and 6.3% in 2006 (p trend = 0.042, p change in trend = 0.277).

### Partners and current marital status

Among those not currently married there was a decrease in the proportion of men reporting ⩾1 partners in the past year from 23.0% in 1996 to 10.0% in 2006 (p trend<0.001, p change in trend = 0.142) in those aged 13–19 years. Among unmarried men aged 20–24 years, the proportion reporting ⩾1 partners in the past year remained fairly stable between 1996 and 2000 (77.0% in 1996 and 74.8% in 2000), but thereafter dropped to 54.3% by 2006 (p trend <0.001, p change in trend = 0.006). Among unmarried women aged 13–19 years there was an overall decline in the percentage reporting ⩾1 sexual partner in the past year, but it initially increased from 17.2% in 1996 to 20.2% in 2000 before decreasing to 12.1% in 2006 (p trend <0.001, p change in trend <0.001). The trend was similar among women aged 20–24 years. It was initially stable between 73.7% in 1996 and 75.0% in 2003 before decreasing to 58.0% in 2006 (p trend = 0.049, p change in trend = 0.076). No significant changes were observed in the other age and sex groups.

Among those currently married there was an increase in the proportion of men aged 25–34 years and 35–44 years reporting ⩾2 partners in the past year between 1996 and 2002, after which the proportions levelled off. Among men aged 25–34 years there was an increase from 27.5% in 1996 to 37.7% in 2002 then to 32.0% in 2006 (p trend = 0.012, p change in trend = 0.014). In married men aged 35–44 years the proportion reporting ⩾2 partners in the past year rose from 15.6% in 1996 to 36.2% in 2002 and then levelled off to 34.4% by 2006 (p trend = 0.001, p change in trend = 0.009). There were no significant changes in the other age and sex groups.

### Condom use

**Figure 3:**
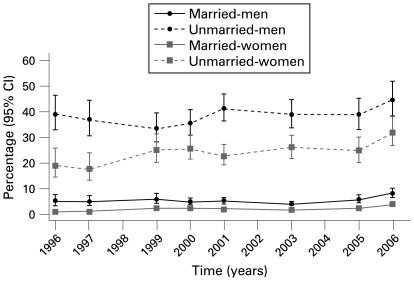
Use of condom at last sex by gender and marital status.

Data on condom use ever were available for the survey years 1993–2006, but on condom use at last sex these data were available for 1996–2006. The proportion of respondents who reported having ever used condoms among those who had ever had sex increased steadily from 7.7% (males 12.5%, females 4.0%) in 1993 to 36.7% (males 46.1%, females 30.7%) in 2006. Condom use at last sex, however, shows a rather different trend. Condom use at last sex among men declined from 18.9% in 1996 to 15.3% in 2000 and 14.7% in 2003, then increased to 15.6% in 2005 and 18.1% in 2006 (p change in trend <0.001). Among women the corresponding figures were 5.7% in 1996 to 8.6% in 2000, followed by a fall to 7.3% in 2001, 8.0% in 2003 and 7.6% in 2005, and finally a rise to 9.9% in 2006 (p change in trend = 0.605, not statistically significant). Both sexes reported declining condom use by age. Married people in this population rarely use condoms (fig 3). Unmarried women were 11.4 times more likely to have used a condom at last sex, while unmarried men were 7.0 times more likely than married persons.

**Figure 4:**
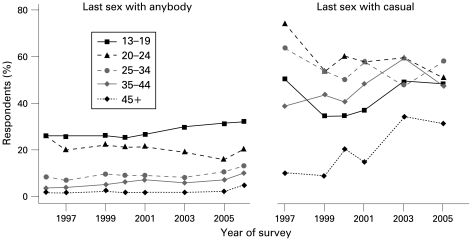
Use of condom at last sex by age of respondent and partnership type.

Condom use with the last casual partner increased among the two oldest age groups (age 35–44 years and 45+ years. It declined in the middle age groups and showed a changing trend (declining and then rising) in 13–19-year-olds (fig 4). Note that condom use at last sex with a casual partner was not asked in as many survey rounds as condom use at last sex with anybody. Among those aged 35–44 years, condom use with the last casual partner rose from 38.9% in 1997 (when this question was first asked) to 47.6% in 2005 (data on this indicator not available for 2006). Among those aged 45+ years it rose from 10.0% in 1997 to 31.3% in 2005. By contrast with the older age groups, the middle age groups experienced a decline in condom use at last sex with a casual partner. The trends in condom use at last sex with a casual partner described here were mainly influenced by men. Condom use with a casual partner among women remained fairly stable over time, in the order of 40% among 13–19-year-olds and generally declining with age.

The percentage of married people who reported at least one casual partner in the past year ranged from 3% to 6%. Condom use at last sex with their casual partner underwent a change in trend over time. Condom use with a casual partner among married people was 67% in 1997, 56% in 1999, 50% in 2000, 57% in 2001, 55% in 2003 and 59% in 2005 (p change in trend = 0.015). The numbers were small so that the changing trend was not statistically significant for either sex, although both sexes showed the same trend. Among married men the percentage who used a condom at last sex with a casual partner was 71% in 1997, 61% in 1999, 59% in 2000, 62% in 2001, 61% in 2003 and 63% in 2005. Although married women were much less likely than men to report a casual partner, those who did were also less likely to use a condom with that partner. Corresponding figures of condom use at last sex with a casual partner among married women were 40% in 1997, 22% in 1999, 8% in 2000, 25% in 2001, 24% in 2003 and 38% in 2005.

**Figure 5:**
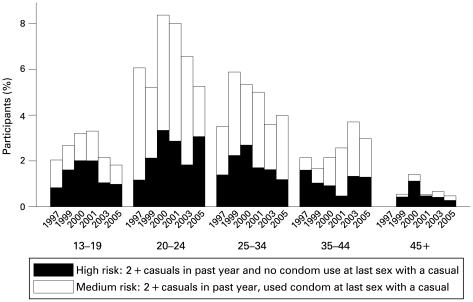
Risk taking behaviour by survey year and age (combining casual partners and condom use).

In some cases, trends in condom use were the opposite of trends in casual partners. For example, among those aged 35+ years, casual partners rose between 2000 and 2005, indicating more risky behaviour, but condom use also rose, indicating less risky behaviour. In order to assess the overall trend in “risky behaviour”, we therefore combined both condom use and casual partners (fig 5). For most age groups risky behaviour—as measured by casual partners and condom use—appears to have risen between 1997 and 2001 and then declined.

## DISCUSSION

This study describes the trends in sexual behaviour from a rural Ugandan cohort during the period 1993–2006. The evolving pattern was not consistent across the different variables investigated and across different subgroups of the population. A summary of the indicators and trends described in this paper is shown in table 2. On the positive side, there was a clear increase in reported median age at first sex for both young men and women and, in line with this, the percentage of young men and women who reported primary abstinence increased. Overall, reported secondary abstinence also increased, albeit more clearly so in young men. Levels of reported condom use at the last sexual act have increased over time and are quite high, particularly among those not currently married reaching, for example, nearly 50% among men aged 20–24 years.

**Table 2:** Summary of direction and magnitude in trends of sexual behaviour

Indicator	Direction of trend	Magnitude	Note
Age at sexual debut		Girls: 16.7–>18.2	Small dip in 1998–2001, same trend for boys and girls
Years 1997–2006	Increasing	Boys: 18.5–>19.9	
Primary abstinence			Dip in 1999–2002 for girls, dip in 1998–2000 for boys
Years 1993–2006	Increasing	72–>83%	
Secondary abstinence			
Years 1997–2006			
Age 13–19	Increasing	Girls: 12–>14%	Same trend for boys and girls
All other ages	No change	Boys: 10–>21%	
Pregnancy			
Years 1996–2003			
Age 13–20	Increasing	8–>10%	Pregnancy among never married young women
Age 21–29	Increasing	53–>81%	
Years 2003–2005			
Age 13–20	Decreasing	10–>6%	
Age 21–29	Decreasing	81–>66%	
Age at first marriage			
Years 1998–2006			
Age 13–29 (women)	Increasing	19.0–>19.4	
Age 13–29 (men)	Increasing	24.9–>25.3	
Time (years) from sexual debut to first marriage			
Years 1998–2006			
Age 13–29 (women)	No change	∼2.2 throughout	
Age 13–29 (men)	Decreasing	7.9–>6.9 years	
⩾1 casual partners in past year			
Years 1997–2001	Increasing	Men: 11.6–>12.6%	Same trend for all groups except men aged 20–24 years and 25–34 years who showed a continuous rise in casual partners throughout the entire period (1997–2006)
		Women: 1.4–>3.6%	
Years 2001–2006	Decreasing	Men: 12.6–>10.2%	
		Women: 3.6–>1.4%	
⩾2 casual partners in past year			Similar trend as for ⩾1 casual partners except that the 35–44 age group showed a continuous increase throughout
Years 1997–2006			
Condom use ever			
Years 1993–2006	Increasing	Men: 12.5–>46.1%	Same trend for men and women, all ages
		Women: 4.0–>30.7%	
Condom use at last sex with any partner			
Years 1996–2003			
Men	Decreasing	18.9–>14.7%	Women: Although a generally rising trend throughout, women experienced a dip in condom use between 2001 and 2005
Women	Increasing	5.7–>8.0%	
Years 2003–2006			
Men	Increasing	14.7–>18.1%	
Women	Increasing	8.0–>9.9%	
Condom use at last sex with a casual			
Years 1997–2005			
Age 20–24	Decreasing	74.1–>50.7%	Among those age 13–19 years, condom use with a casual decreased from 1997 to 2000, then increased from 2000 to 2005
Age 25–34	Decreasing	63.8–>58.2%	
Age 35–44	Increasing	38.9–>47.6%	
Age 45+	Increasing	10.0–>31.3%	
Age 13–19	Decrease–>Increase	50.6–>48.2%	

On the other hand, the percentage of young women aged 13–19 years who experienced pregnancies before marriage was rather high at around 7% and showed little change over time. The percentage of never married women aged 21–29 years who became pregnant increased from 53% to nearly 70% and started to fall somewhat only by 2004. Both observations suggest that sexual risk taking among young women may have increased during the observation time. With respect to casual partners, although total numbers were low, the percentage of women reporting sexual intercourse with one or more such partners during the last 12 months tripled between 1997 and 2004, a trend that has only slowly reversed thereafter. Casual sex was much more often reported by men. The trend among men was similar to that reported by women over time, although the increase until 2004 was much weaker. Perhaps most important is the observation that condom use with casual partners showed a declining trend over time for these age bands and by 2005 no longer exceeded 60%, as had been the case by 1997. Interestingly, condom use with casual partners rose steeply over time in those aged 35–44 years and ⩾45 years, with men contributing most of the data. All in all, the evolving picture is a mixed one.

Several of our observations mirror developments in other parts of Uganda and in some other African countries. The encouraging increase in the age of sexual debut continues the trend reported during the early 1990s in Uganda.^3^ ^6^ This increase has also been observed in Zimbabwe, Kenya and Ghana.^5^ ^17^ An increase in secondary abstinence has been noted among women from 18 AIDS-affected African countries during the 1990s, during which time the median percentage of women reporting no sexual activity during the past 3 months rose from 44% to 49%, and rose significantly in 7 out of 18 countries.^18^ Condom use has reached high levels among young sexually active people elsewhere in Uganda. In a study of school-going youth in western Uganda, ever use of condoms was reported to have increased from 50% in 1996 to 73% in 2001,^19^ and 80% of school-going young men and 72% of young women in urban areas reported condom use at the last sexual act in 2003.^20^ Again, this reinforces a development that had already begun in Uganda before 1997.^21^

The worrying levels of sexual relationships with casual partners and observations about insufficient condom use with such partners have also been observed elsewhere in Uganda and Africa. In a recent study, 51% of men and 32% of women among the work force of a sugar factory in southern Uganda reported multiple sex partners in the past year, and only 36% of the respondents reported condom use for the last sex act with a casual partner.^22^ High levels of unprotected sex with multiple partners have been reported recently in other countries in southern Africa such as Botswana.^23^

Not all countries have observed similar trends to Uganda. For example, in a cohort from Tanzania followed from 1995 to 2000, most individuals felt that they were not at risk from HIV infection and sexual risk behaviour remained largely unchanged.^24^

The question arises whether the trends in reported sexual behaviour may help to explain recent changes in the trend of the HIV epidemic. Our sexual behaviour data are from the same population in which we documented that, since about the year 2000, the prevalence and incidence of HIV were no longer falling.^25^ The data reported in the paper presented here do not link reported sexual risk factors and incident HIV infection. However, we noted that a number of temporary or maintained decreases in protective behaviour occurred during the period from 1998 to 2002. For example, while primary abstinence among youth aged 13–19 years generally increased over time, there was a short period between 1998 and 2002 during which it dipped. Changes in other indicators also seem to be consistent with the increase in transmission risk during that period, such as increases in reported pregnancy among unmarried young women, temporary declines in age at sexual debut and temporary declines in reported secondary sexual abstinence and condom use with casual partners (eg, in the youngest age group). The proportion of both men and women who reported a casual partner in the past year rose steadily from 1997 to 2001. In the period between 1996 and 2002 there was an increase in the proportion of married men in the 25–34 years and 35–44 years age groups reporting ⩾2 partners in the past year. The proportion who reported casual partners in these two age groups continued to rise until 2004, when the incidence was rising as a whole, and a second peak of incidence was also observed among women in their 30s and men in their 40s.^25^ However, while these observations are sufficiently strong to inform public health and policy and call for a renewed emphasis on prevention efforts in Uganda, definite conclusions regarding a link between observed sexual behaviour and epidemiological trends are not yet possible.

In addition to previously identified public health messages, the results of our study point to the need for a mix of group-specific prevention efforts. For example, our data show that risky behaviour may be increasing among older people, and this has coincided with an increase in the prevalence and incidence of HIV in this group. At the same time, condom use (even with casual partners) consistently declined with increasing age, creating a prevention gap that needs to be addressed.

At the advanced stage of the epidemic in which Uganda and most sub-Saharan African countries now fall, most HIV transmissions occur within stable partnerships.^26^ ^27^ For a variety of reasons, condom use within marriages is very low, even among HIV-discordant couples.^28^^–^30 In many parts of Africa married couples find it difficult to accept condom use.^30^ For example, while the national survey of 2004 in Uganda showed that protected sex within marriages occurred three times more often among infected partners who knew their status than among those who did not, condom use was still low.^1^ In addition to the traditional focus on avoiding extramarital sexual contacts, prevention programmes in mature epidemics should pay greater attention to HIV protection *within* stable partnerships. Barriers to couple testing^31^ ^32^ in Africa have been well documented. Raising awareness of the issue of serodiscordance might remove one of the most important barriers to HIV testing^33^ by challenging the notion that mutual infection is inevitable once one couple member becomes infected. It is, however, likely that, in spite of all efforts, condom use in marriage will remain low even among discordant couples who know their HIV status. Alternative intervention strategies are needed. If shown to be effective, these may include microbicides, post-exposure prophylaxis, early initiation of antiretroviral therapy (ART), suppression of herpes simplex virus-2 infection and, ultimately, a vaccine against HIV infection.

### Limitations and possible biases

Our study includes some possible biases. Just 57% of the population who participated in the census provided information on sexual behaviour in any given round. Individuals who did not respond may have higher risk taking behaviour. However, the bias is unlikely to have changed over time, so observed trends should not be affected. Also, some questions were not always asked the same way and this may have introduced bias. For example “ever had sex” was a direct question in 1993 but, from 1997, it was derived from the reported age at first sex.

Another possible bias regards data quality. Interviews were administered face-to-face. During the consent process the respondents were reassured of confidentiality of the interviews; however, some social desirability bias may exist. In addition, we used open-ended classification while obtaining responses on number of partners and may therefore have failed to identify those very few individuals who have very large numbers of partners. Men report much higher numbers of casual partners and also higher condom use than women. Yet, in this comparatively closed society, these findings are questionable. With regard to partnership type, the difference in reporting could be a question of perception of partnership type as women and men may have different perceptions about the seriousness of partnerships.^34^ It is most likely that men over-report while women under-report, a phenomenon observed in most sexual behaviour surveys.^35^ An interpretation that would make the reporting trends between men and women consistent is that there may be some women who have many sexual partners while most have just one regular partner. The data do not justify this interpretation, however, as 1.12% of men over time have claimed ⩾5 casual partners in the past year and 1.68% have claimed ⩾5 partners of any type, while only 0.02% of women have claimed ⩾5 casual partners and 0.06% have claimed ⩾5 partners of any type.

It is unclear why some people in this population began engaging in more risky sexual behaviour. Some researchers have suggested that “normalisation” may have contributed to this, with HIV now becoming a normal part of life, possibly leading to complacency.^36^ We believe it unlikely that the introduction of ART influenced trends in sexual behaviour in this study. If anything, after a period of increased risky behaviour, risky sexual behaviour appears to be declining in 2004/5, at the time that ART was introduced. Also, by 2006, only about 20% of the HIV-positive respondents were receiving ART. So far, several studies from Uganda seem to indicate that the availability of ART has not introduced disinhibition of sexual behaviour, either among ART recipients or in the general population.^37^

## CONCLUSION

In this rural cohort we observed a number of changes in sexual behaviour between 1993 and 2006. The main findings were:

- The changes in sexual behaviour were not consistent across the variables and subgroups investigated.
- Some indicators showed encouraging trends towards safer sexual behaviour, particularly among young people.
- Other indicators suggest an increase in sexual risk taking, particularly among middle-aged and older adults.
- These changes may help explain recent changes in epidemiological trends in Uganda, but more work is required to establish such links if they exist.

While the reasons for the observed behaviour changes are not well understood, our observations suggest that HIV prevention messages should be reinforced and should be adapted to the needs of particular subgroups. Even though we are more than 20 years into the epidemic, and even though Uganda was a leader in promoting public health awareness and prevention of HIV throughout the 1990s, we must not relax.
